# Supplementary figures and images for: Embedding optimization reveals long-lasting history dependence in neural spiking activity
Source: PLoS Comput Biol. 2021 Jun 1;17(6):e1008927. doi: 10.1371/journal.pcbi.1008927 (PMC8205186; doi:10.1371/journal.pcbi.1008927)

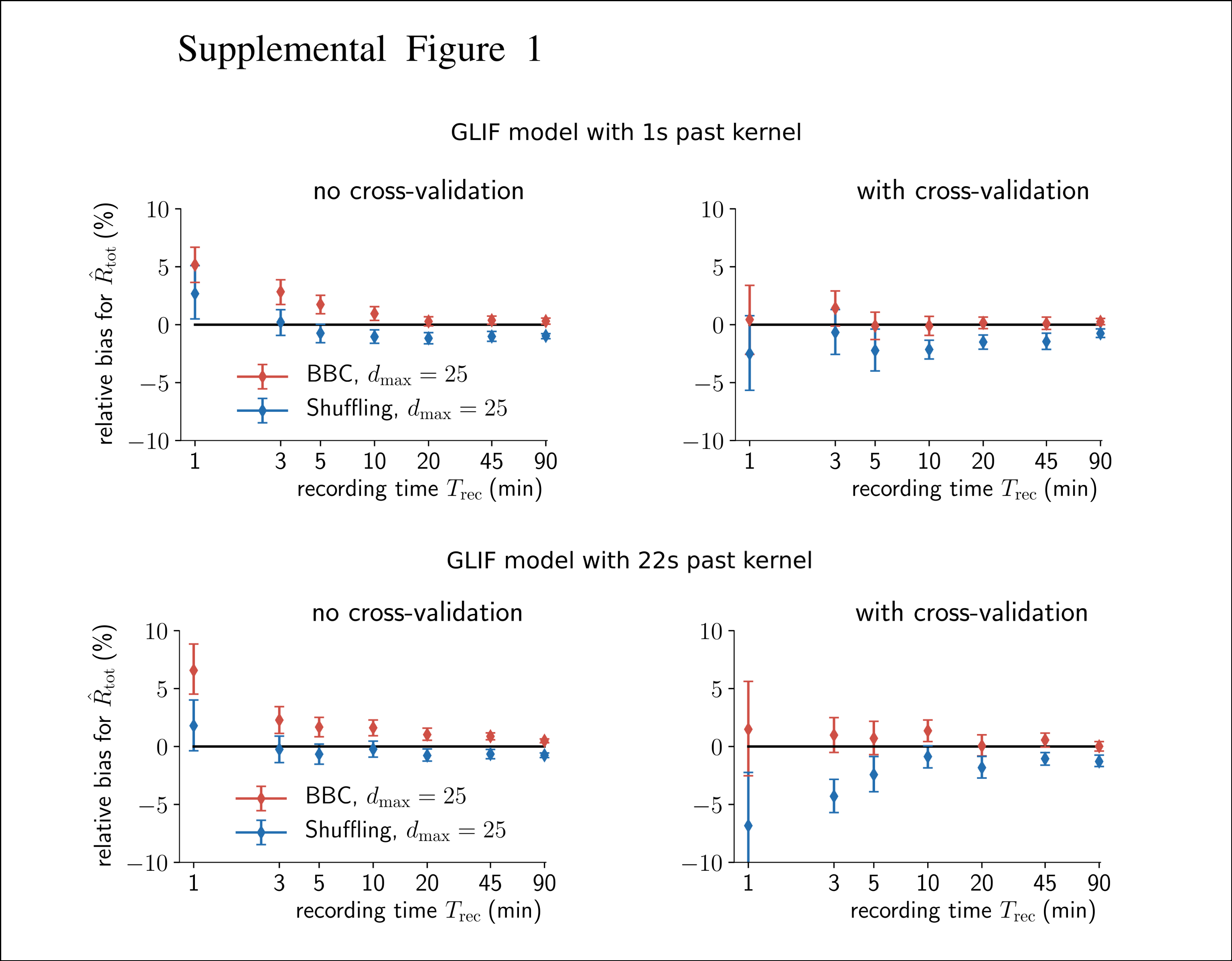

Supplement: S1 Fig — Shown is the relative bias for two versions of the GLIF model with spike adaption, one with 1s and the other with 22s past kernel. The relative bias refers to the relative difference between embedding-optimized estimates R^(T,d*,κ*) and the model’s true history dependence R(T, d*, κ*) for the same optimized embedding parameters d*, κ*. The relative bias for R^tot was computed by first averaging the relative difference (R^(T,d*,κ*)−R(T,d*,κ*))/R(T,d*,κ*) for T ∈ [TD, Tmax], and second averaging again over 30 different simulations for Trec between 1 and 20 minutes, and 10 different simulations for 45 and 90 minutes. Embedding parameters were optimized for each simulation, respectively, using parameters as in Table 2 with dmax = 25. (Left) For BBC, the relative bias for R^tot is zero only if recordings are sufficiently long (> 20 minutes for 1s kernel, and ≈ 90 minutes for 22s kernel). When recordings are shorter, the relative bias increases, and thus estimates are mildly overestimating the model’s true history dependence for the optimized embedding parameters. For Shuffling, estimates provide lower bounds to the model’s true history dependence, hence the relative bias remains negative even in the presence of overfitting. (Right) When one round of cross-validation is applied, i.e. embedding parameters are optimized on the first, and estimates are computed on the second half of the data, the bias is approximately zero for BBC even for short recordings, or more negative for the Shuffling estimator. Therefore, we conclude that the origin of overfitting is the selection of embedding parameters on the same data that are used for the estimation of R. Errorbars show 95% bootstrap confidence intervals on the mean over n = 10 (45 or 90 min) or n = 30 (≤ 20 min) different simulations. (TIF) [file pcbi.1008927.s001.tif]

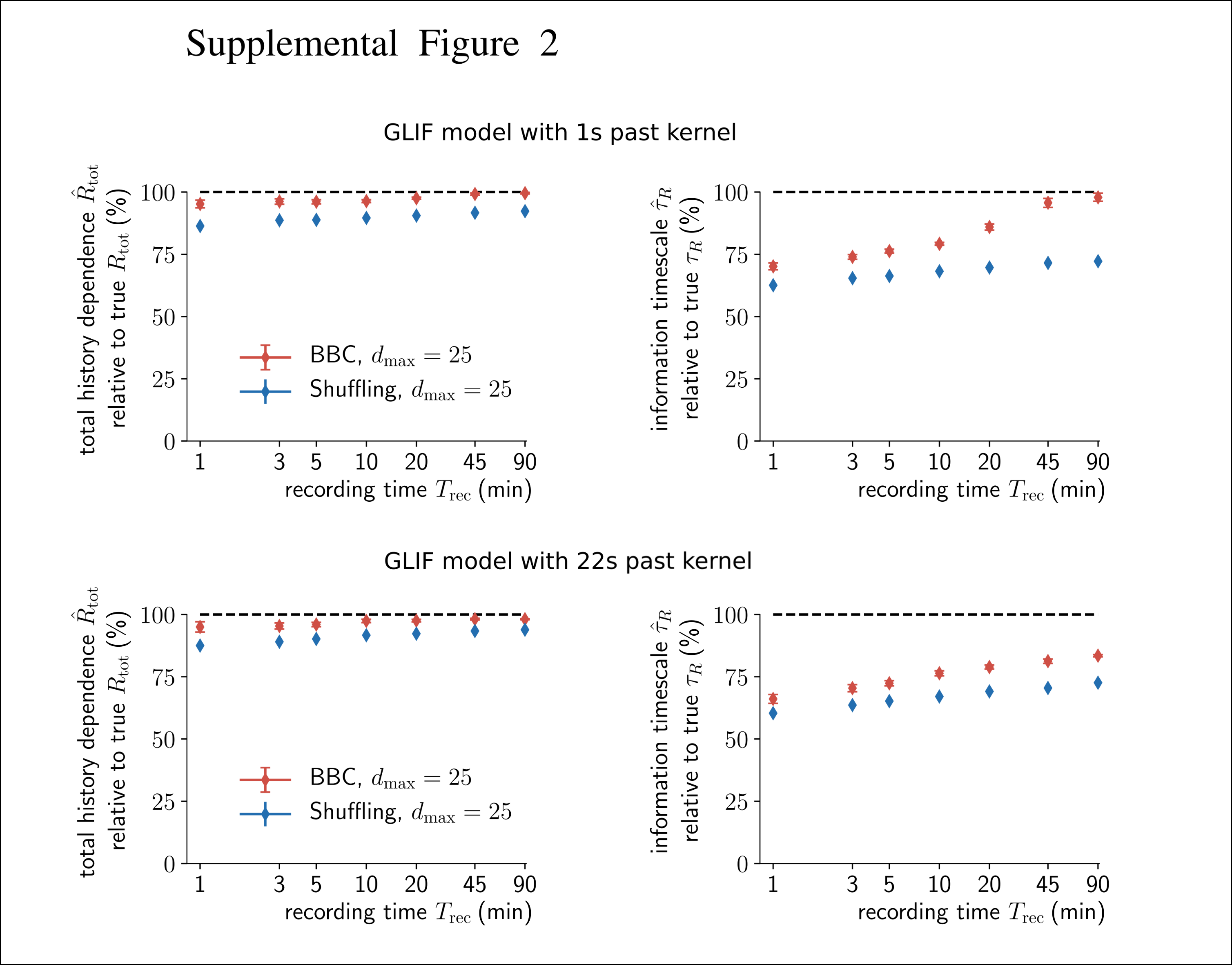

Supplement: S2 Fig — (Left) Mean estimated total history dependence R^tot for different recording lengths, relative to the true total history dependence Rtot of the model (GLIF with spike adaption with 1s or 22s past kernel). As the recording length decreases, so does R^tot. However, with only 3 minutes, one does still infer about ≈ 95% of the true Rtot. (Right) In contrast, the estimated information timescale τ^R decreases strongly with decreasing recording length. With 3 minutes and less, only ≈ 75% of the true τR is estimated on average. Note that for the simpler 1s model (top), an accurate estimation of the true τR is possible for 90 minute recordings, whereas for the 22s model (bottom), the estimated τ^R remains below the true value. Shown are mean values for 30 different simulations for Trec between 1 and 20 minutes, and 10 different simulations for 45 and 90 minutes, as well as 95% confidence intervals on the mean based on bootstrapping. (TIF) [file pcbi.1008927.s002.tif]

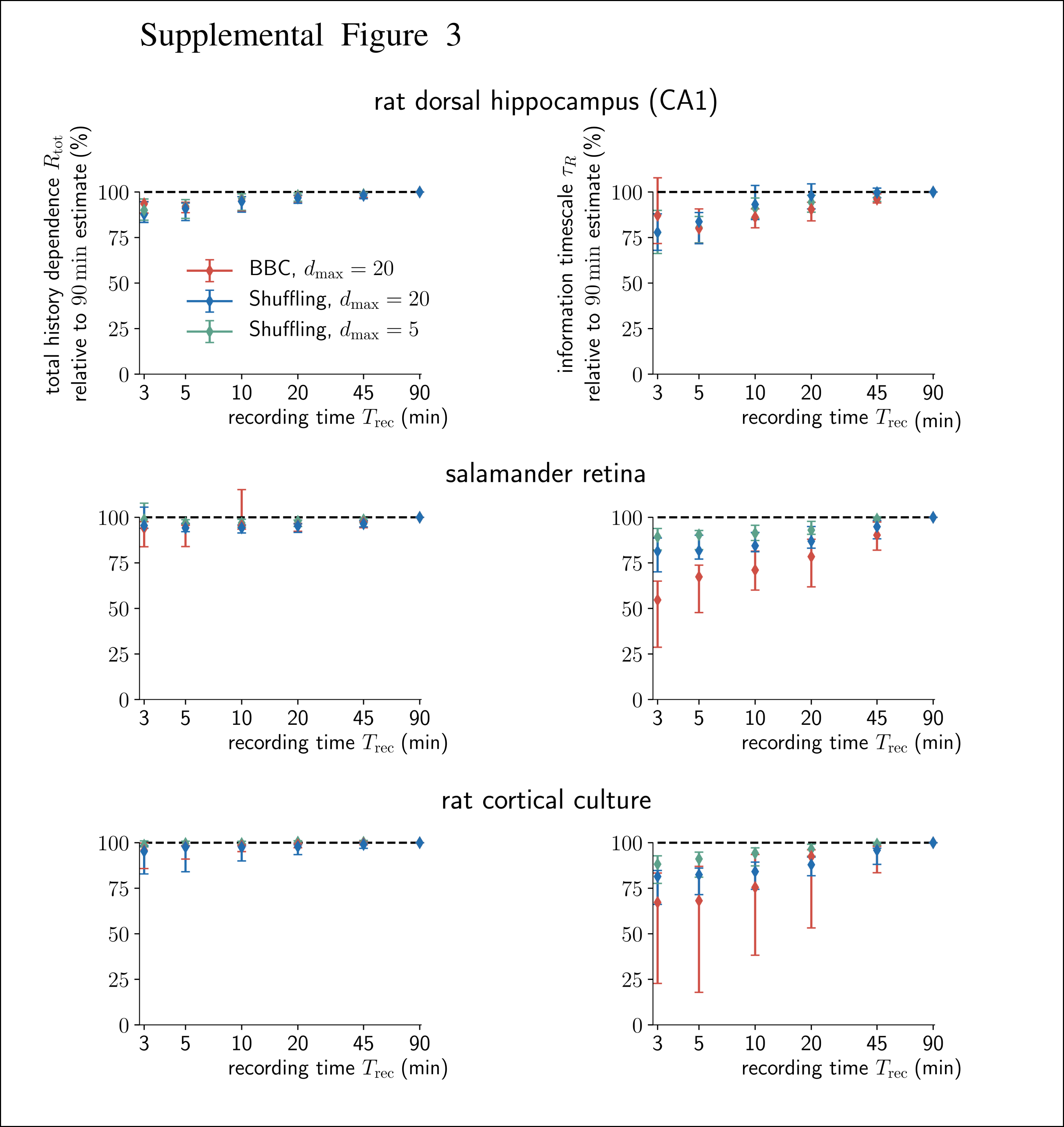

Supplement: S3 Fig — (Left) Total history dependence Rtot for different recording lengths, relative to the total history dependence estimated for a 90 minute recording. As long as recordings are 10 minutes or longer, one does still estimate about ≈ 95% as much or more of Rtot as for 90 minutes, for all three recordings. For less than 10 minutes, the estimated total history dependence decreases down to 90% (CA1), or increases again due to overfitting (retina). (Right) Similar to the GLIF model, the estimated information timescale τR decreases more strongly with decreasing recording length. With 10 minutes and more, one estimates around ≈ 75% or more of the τR that is estimated on a 90 minute recording. Note that for the experimental data, the estimated timescale of the BBC estimator depends more strongly on the recording time, whereas the Shuffling estimator is more robust, especially for dmax = 5. Shown is the median with 95% bootstrap confidence intervals over n = 10 randomly chosen sorted units for each neural system. Before taking the median over sorted units, for each unit we averaged estimates over 10 excerpts of the full recording, each with 3 or 5 minutes duration, and over 8,4 and 2 excerpts with 10, 20 and 45 minutes duration, respectively. (TIF) [file pcbi.1008927.s003.tif]

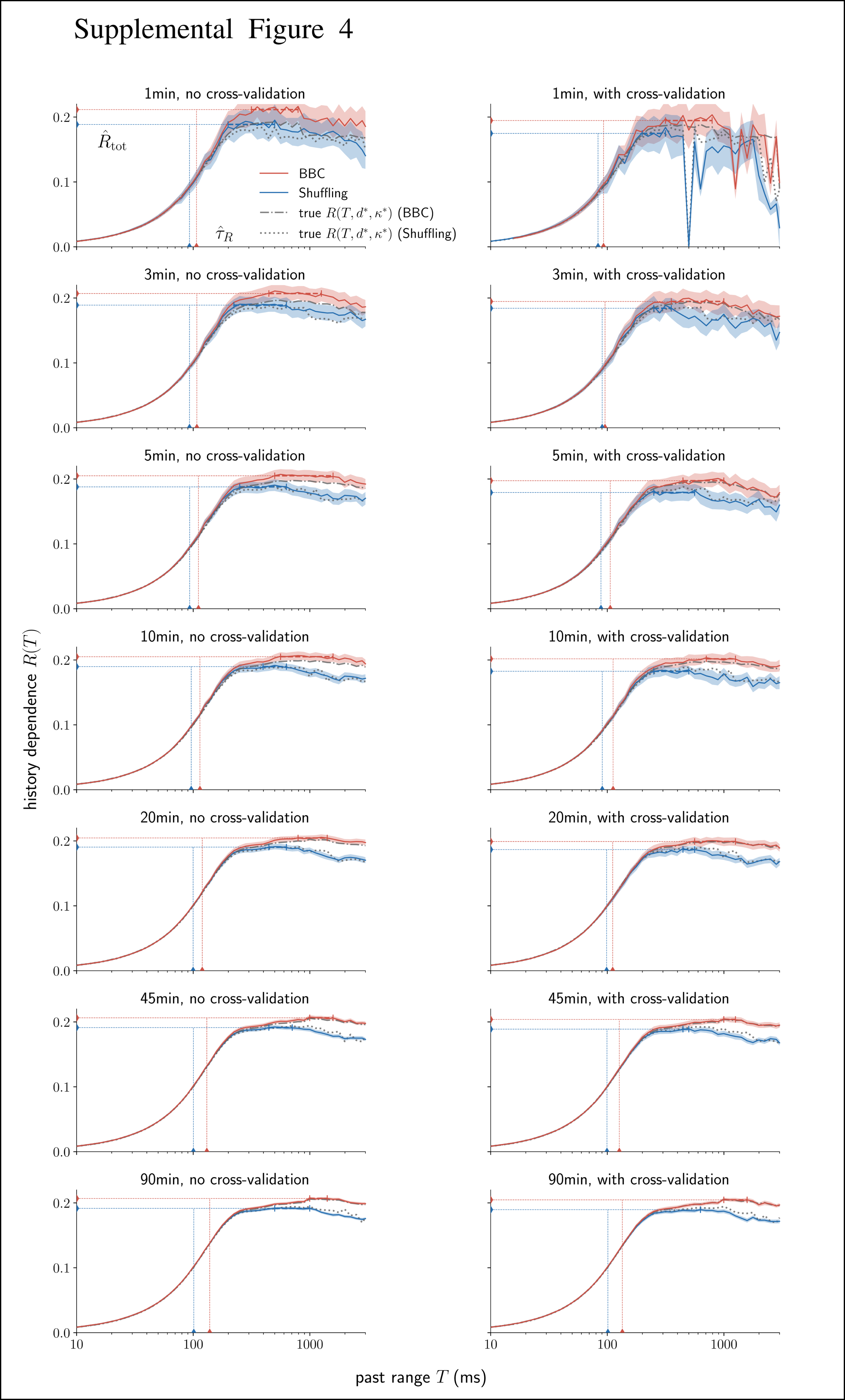

Supplement: S4 Fig — For each recording length, we show the embedding-optimized estimates of history dependence R(T) with and without cross-validation, for BBC (red) and Shuffling (blue) with dmax = 25, as well as the ground truth for the same embeddings that were found during optimization (dashed lines). Dashed lines indicate the estimated information timescale τ^R and total history dependence R^tot. Shaded areas indicate ± two standard deviations obtained by bootstrapping. (TIF) [file pcbi.1008927.s004.tif]

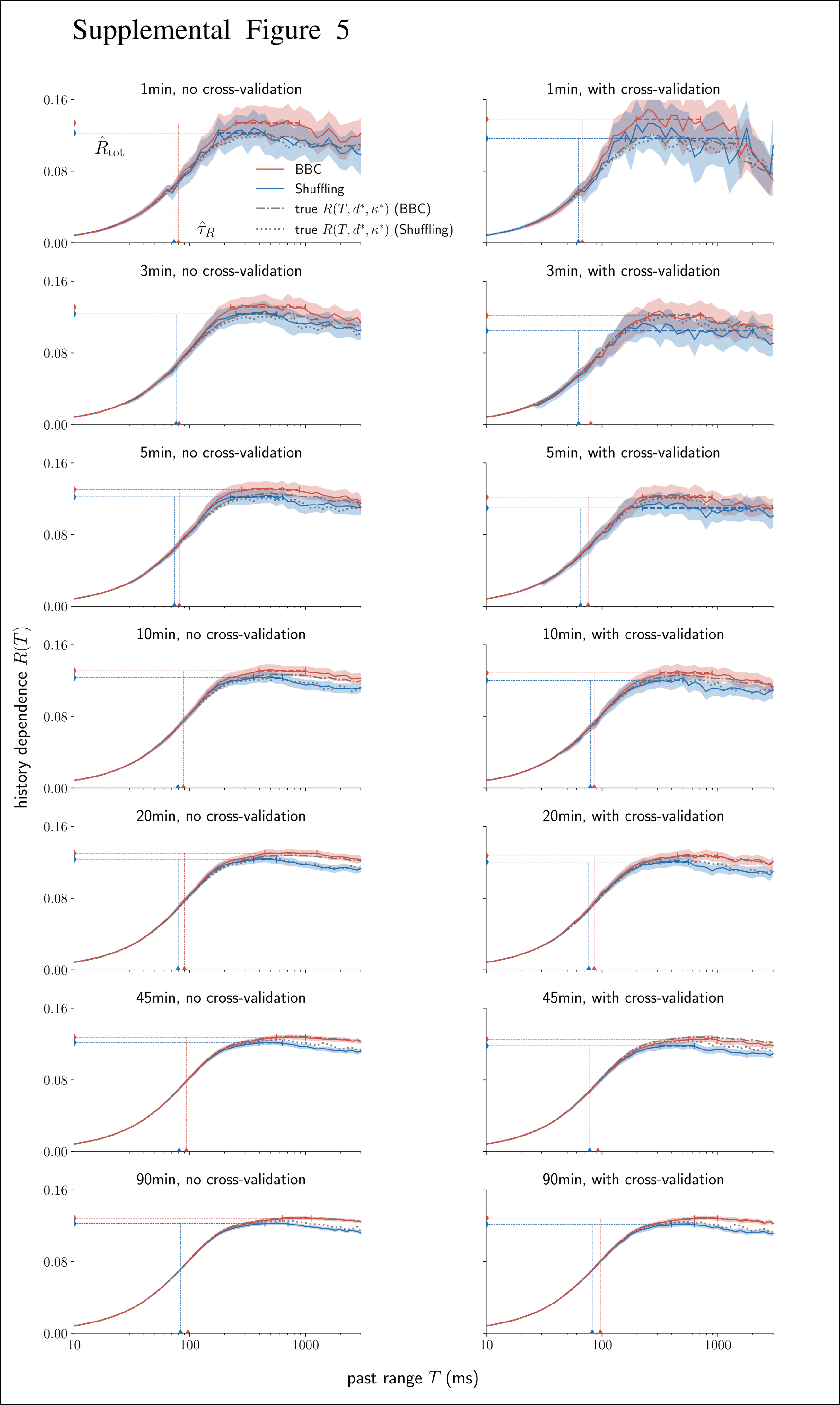

Supplement: S5 Fig — For each recording length, we show the embedding-optimized estimates of history dependence R(T) with and without cross-validation, for BBC (red) and Shuffling (blue) with dmax = 25, as well as the ground truth for the same embeddings that were found during optimization (dashed lines). Dashed lines indicate the estimated information timescale τ^R and total history dependence R^tot. Shaded areas indicate ± two standard deviations obtained by bootstrapping. (TIF) [file pcbi.1008927.s005.tif]

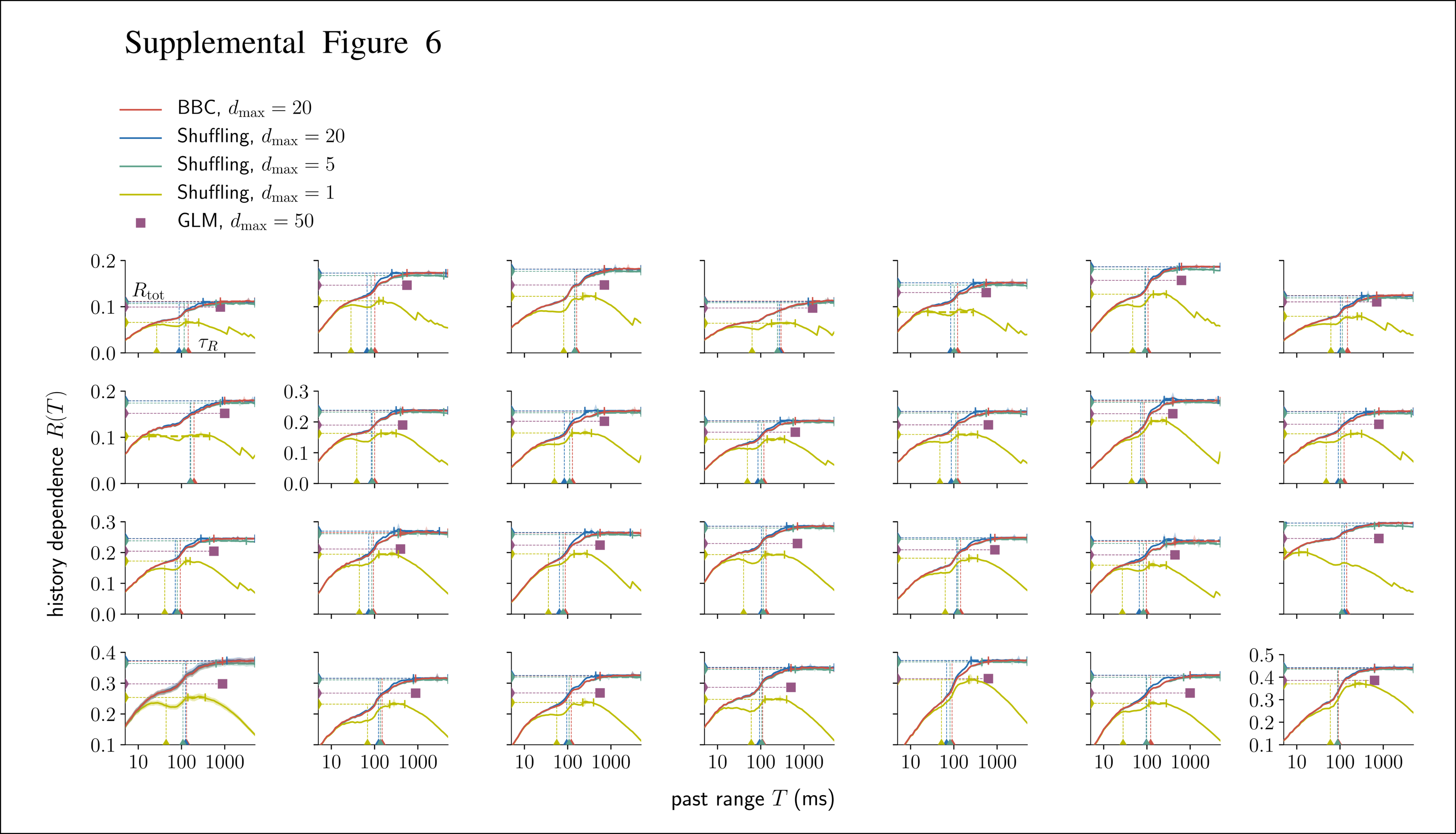

Supplement: S6 Fig — For each unit, we show the embedding-optimized estimates of history dependence R(T) for BBC with dmax = 20 (red), as well as Shuffling with dmax = 20 (blue), dmax = 5 (green) and dmax = 1 (yellow). Dashed lines indicate estimates of the information timescale τR and total history dependence Rtot. Also shown is the embedding-optimized GLM estimate (violet square) with a past range equal to the temporal depth that was found with the BBC estimator. (TIF) [file pcbi.1008927.s006.tif]

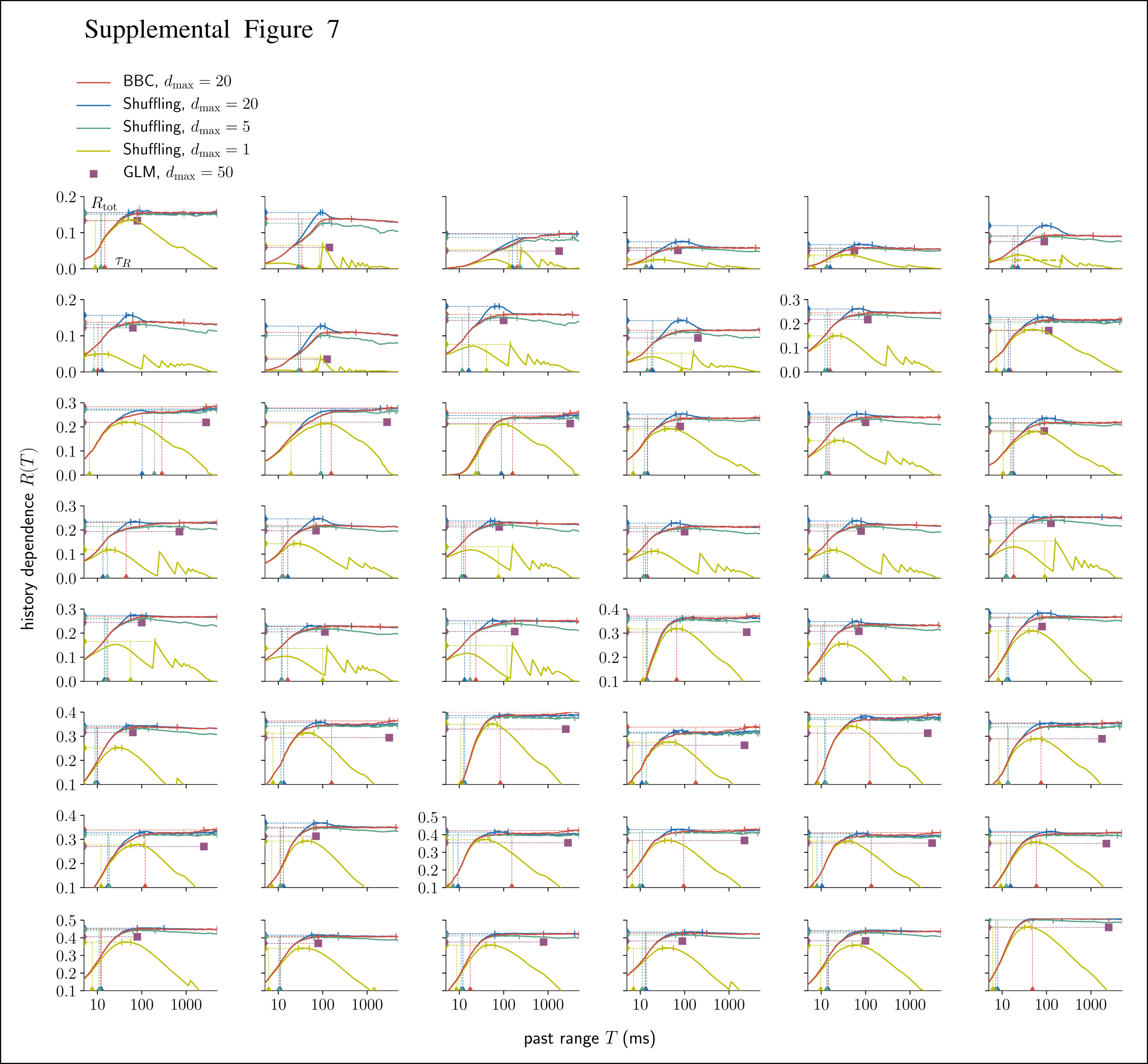

Supplement: S7 Fig — For each unit, we show the embedding-optimized estimates of history dependence R(T) for BBC with dmax = 20 (red), as well as Shuffling with dmax = 20 (blue), dmax = 5 (green) and dmax = 1 (yellow). Dashed lines indicate estimates of the information timescale τR and total history dependence Rtot. Also shown is the embedding-optimized GLM estimate (violet square) with a past range equal to the temporal depth that was found with the BBC estimator. (TIF) [file pcbi.1008927.s007.tif]

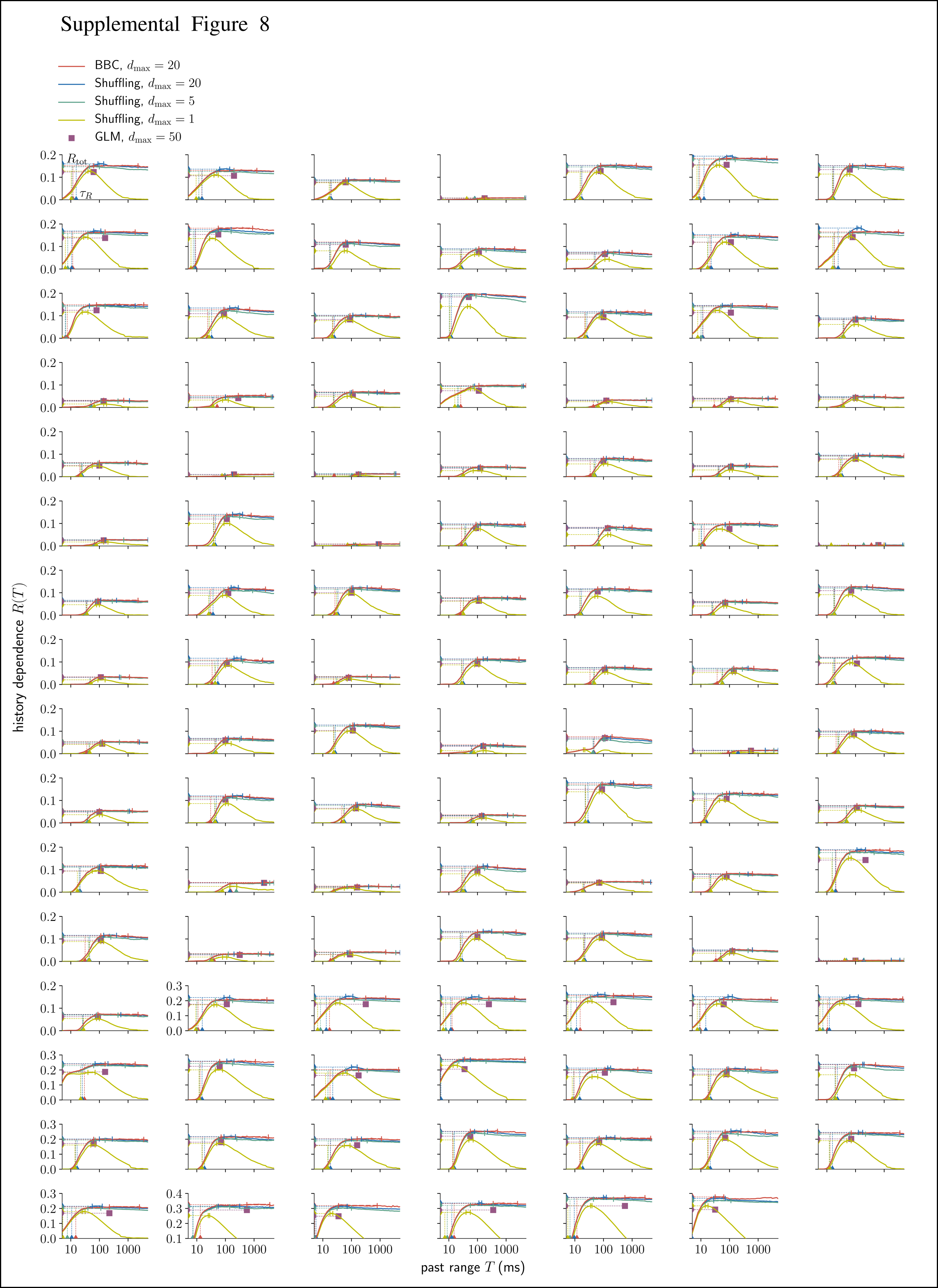

Supplement: S8 Fig — For each unit, we show the embedding-optimized estimates of history dependence R(T) for BBC with dmax = 20 (red), as well as Shuffling with dmax = 20 (blue), dmax = 5 (green) and dmax = 1 (yellow). Dashed lines indicate estimates of the information timescale τR and total history dependence Rtot. Also shown is the embedding-optimized GLM estimate (violet square) with a past range equal to the temporal depth that was found with the BBC estimator. (TIF) [file pcbi.1008927.s008.tif]

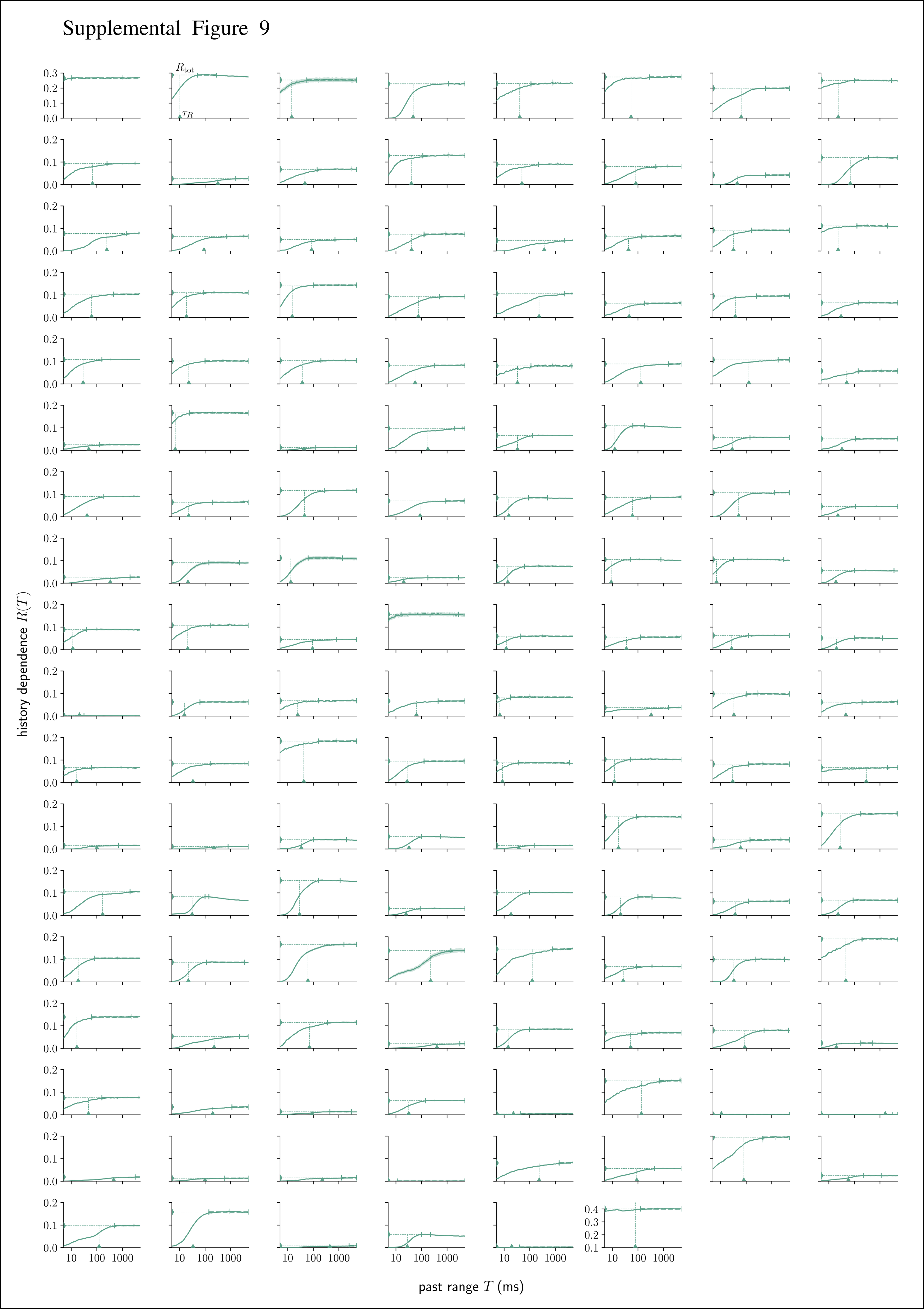

Supplement: S9 Fig — For each unit, we show the embedding-optimized Shuffling estimates of history dependence R(T) for dmax = 5. Dashed lines indicate estimates of the information timescale τR and total history dependence Rtot. (TIF) [file pcbi.1008927.s009.tif]

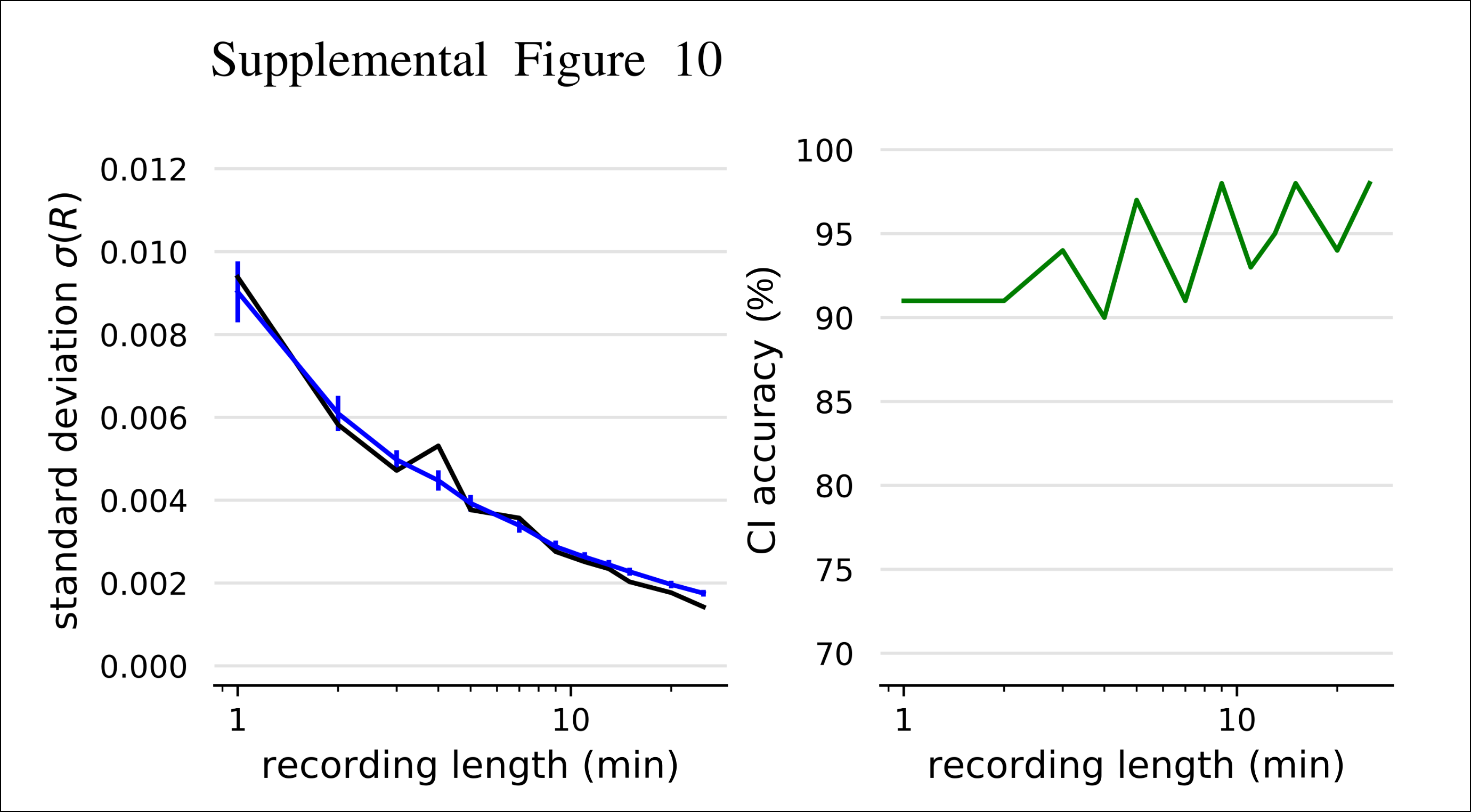

Supplement: S10 Fig — (Left) Shown is the standard deviation on BBC estimates (blue) obtained from 250 “blocks of blocks” bootstrap samples on a single recording (GLIF model with 22s past kernel). It agrees well with the true standard deviation (black), which we estimated from 100 repeated simulations of the same recording length and embedding. As expected, the standard deviation decreases substantially for longer recordings. For each recording length, estimates were computed for typical optimal embedding parameters d*, κ* and T = TD that were found by embedding optimization. Errorbars show mean and standard deviation of the estimated σ(R) over the repeated simulations. (Right) The 95% confidence intervals based on two standard deviations σ(R) have approximately the claimed confidence level (CI accuracy). Standard deviation was estimated from 250 “blocks of blocks” bootstrap samples. For each recording length, we computed estimates R^ and the bootstrap confidence intervals on the 100 simulations. We then computed the confidence level (CI accuracy) by counting how often the true value of R was contained in the estimated confidence interval (green line). Estimates and the true value of R were computed for the same typical embedding parameters d*, κ* and T = TD as before. (TIF) [file pcbi.1008927.s010.tif]

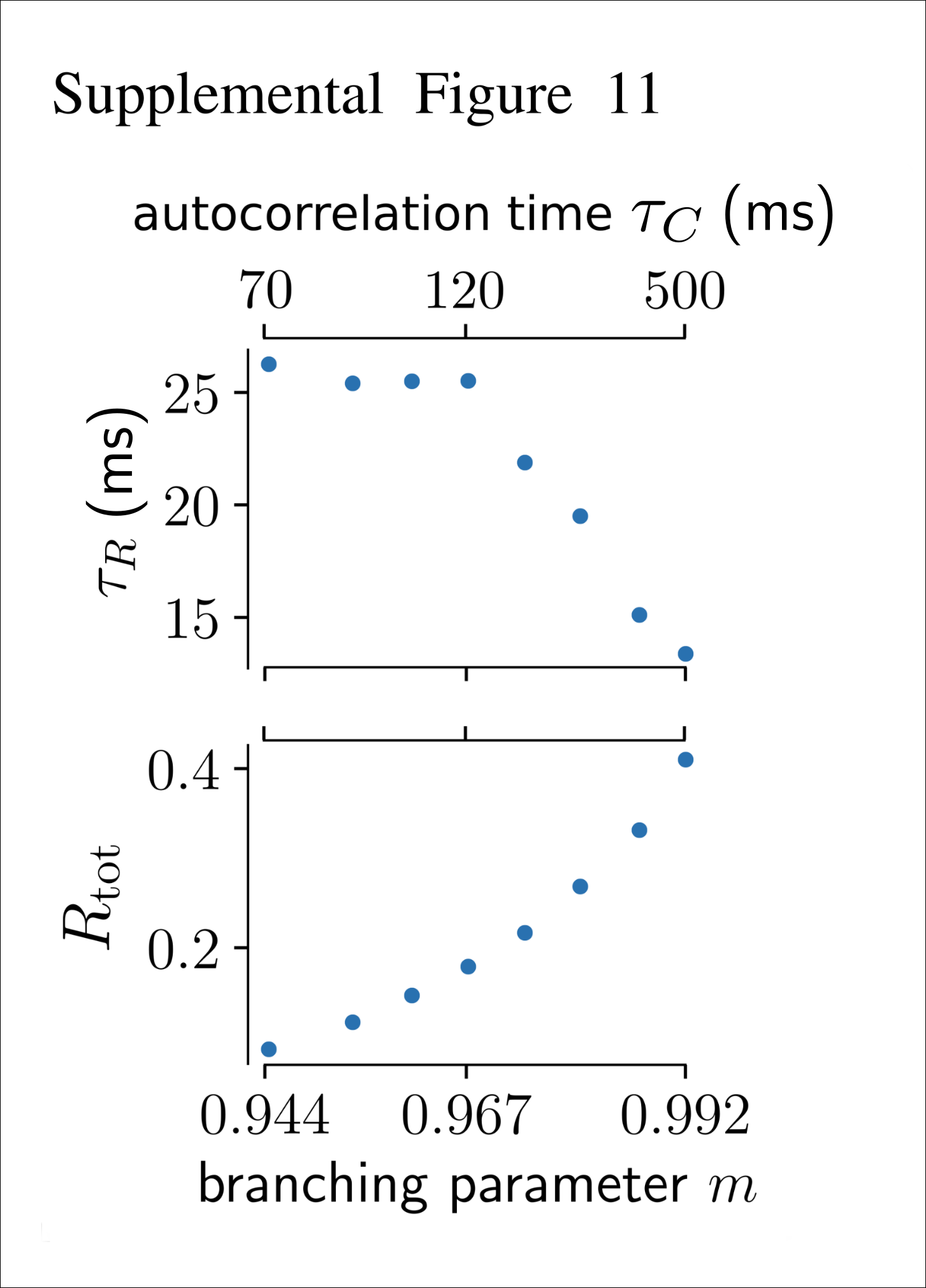

Supplement: S11 Fig — Similar to the binary autoregressive process, increasing the branching parameter m increases the total history dependence Rtot, whereas the information timescale τR stays constant, or even decreases for high m. For each m, the input activation probability h was adapted to hold the firing rate fixed at 5 Hz. (TIF) [file pcbi.1008927.s011.tif]

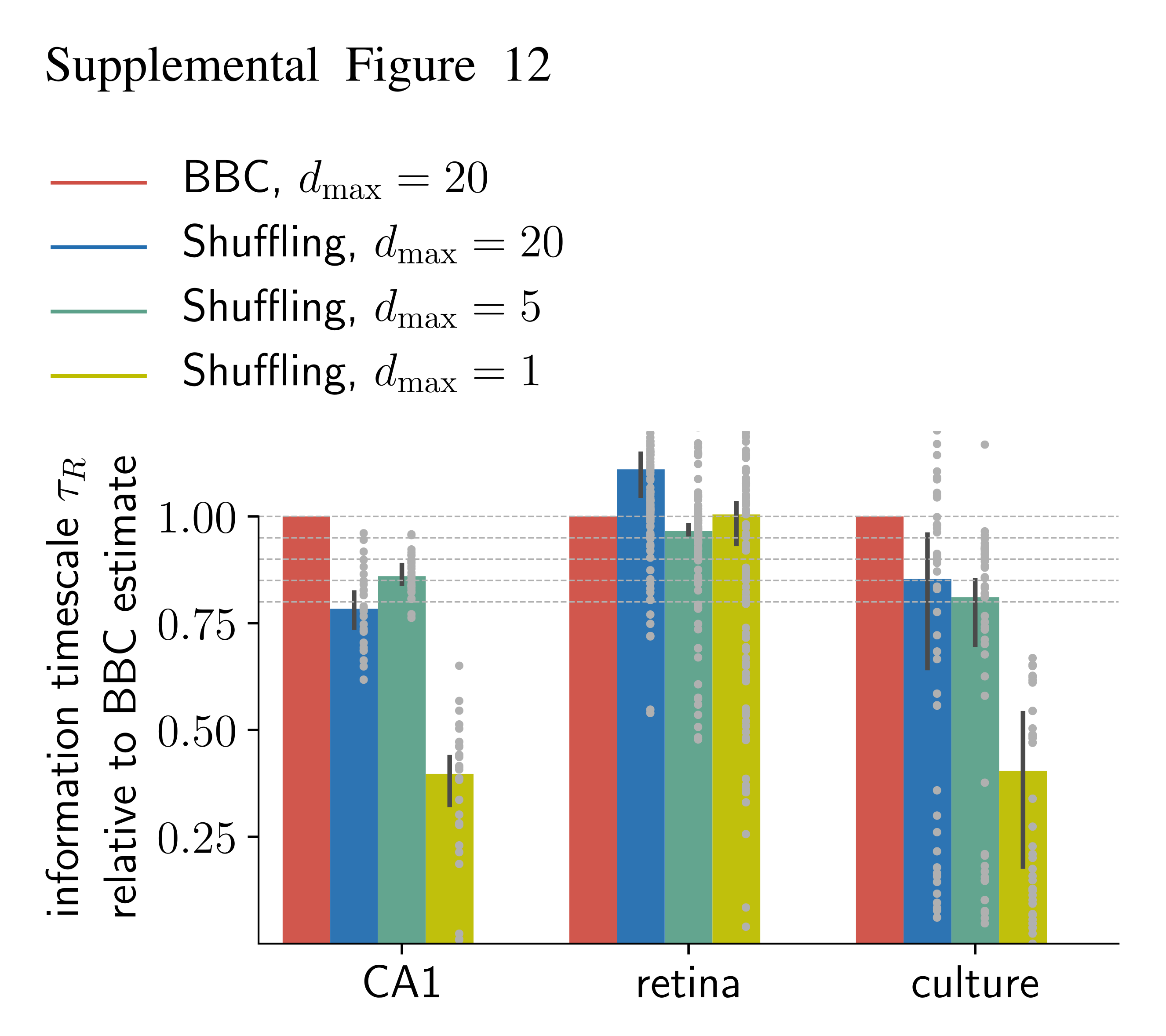

Supplement: S12 Fig — For each sorted unit (grey dots), estimates of the information timescale τR are plotted relative to the corresponding BBC estimate for dmax = 20. The BBC estimator tends to estimate higher timescales than the Shuffling estimator on recordings of CA1 and cortical culture, whereas for retina the medians of different estimators are more similar. Although estimates of the timescale are highly variable between estimators, Shuffling with only dmax = 5 past bins still estimates timescales of at least 80% of the timescales that are estimated with BBC. Errorbars indicate median over sorted units and 95% bootstrap confidence intervals on the median. (TIF) [file pcbi.1008927.s012.tif]

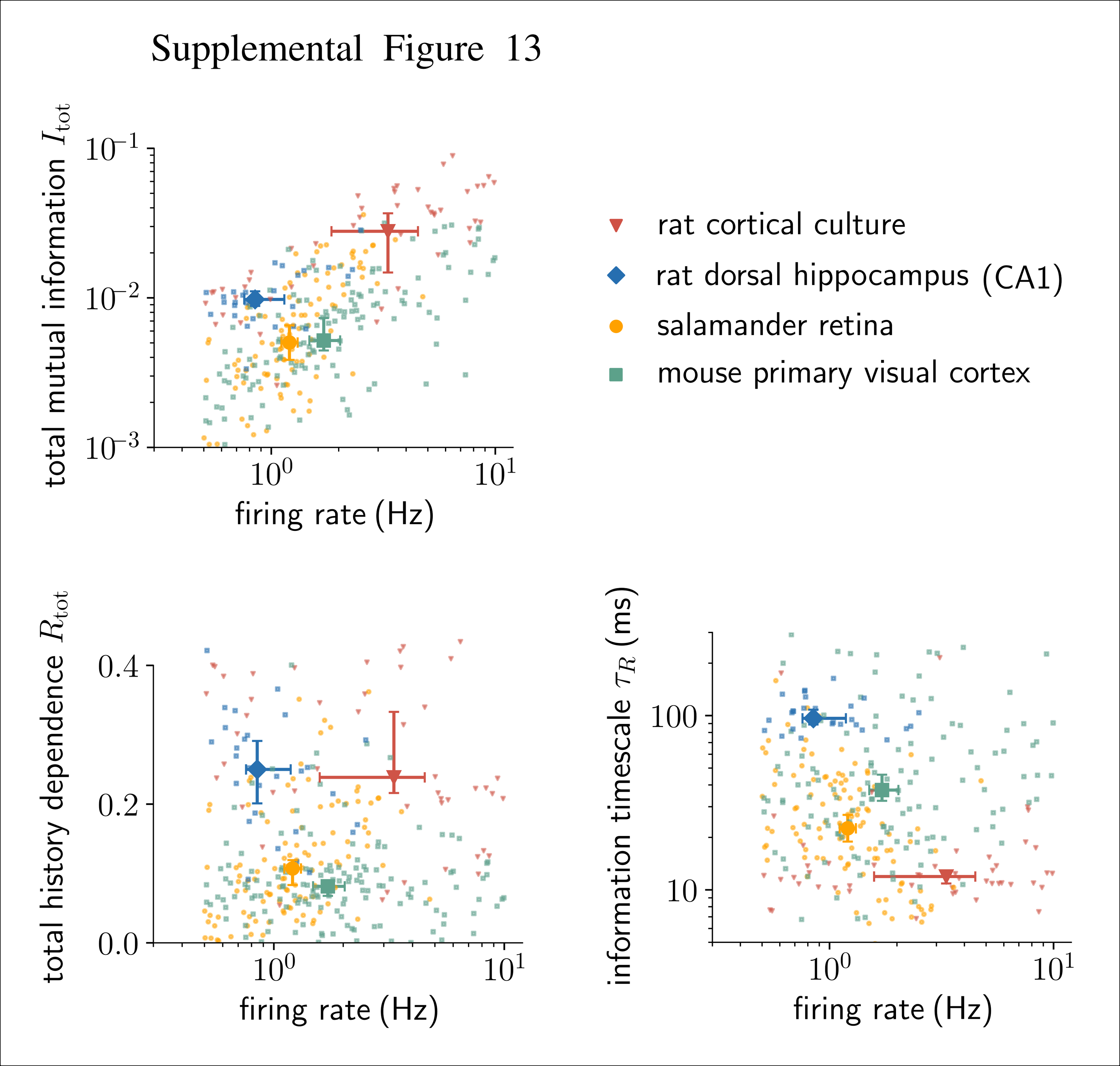

Supplement: S13 Fig — Shown are the same estimates of the total history dependence Rtot and information timescale τR as in Fig 7 (Shuffling estimator with dmax = 5) versus the firing rates of sorted units (dots). The total mutual information Itot is equal to Rtot times the spiking entropy H(spiking) of the respective unit. While Itot tends to increase with firing rate, no clear relation is visible for Rtot or τR. Errorbars indicate median over sorted units and 95% bootstrap confidence intervals on the median. (TIF) [file pcbi.1008927.s013.tif]

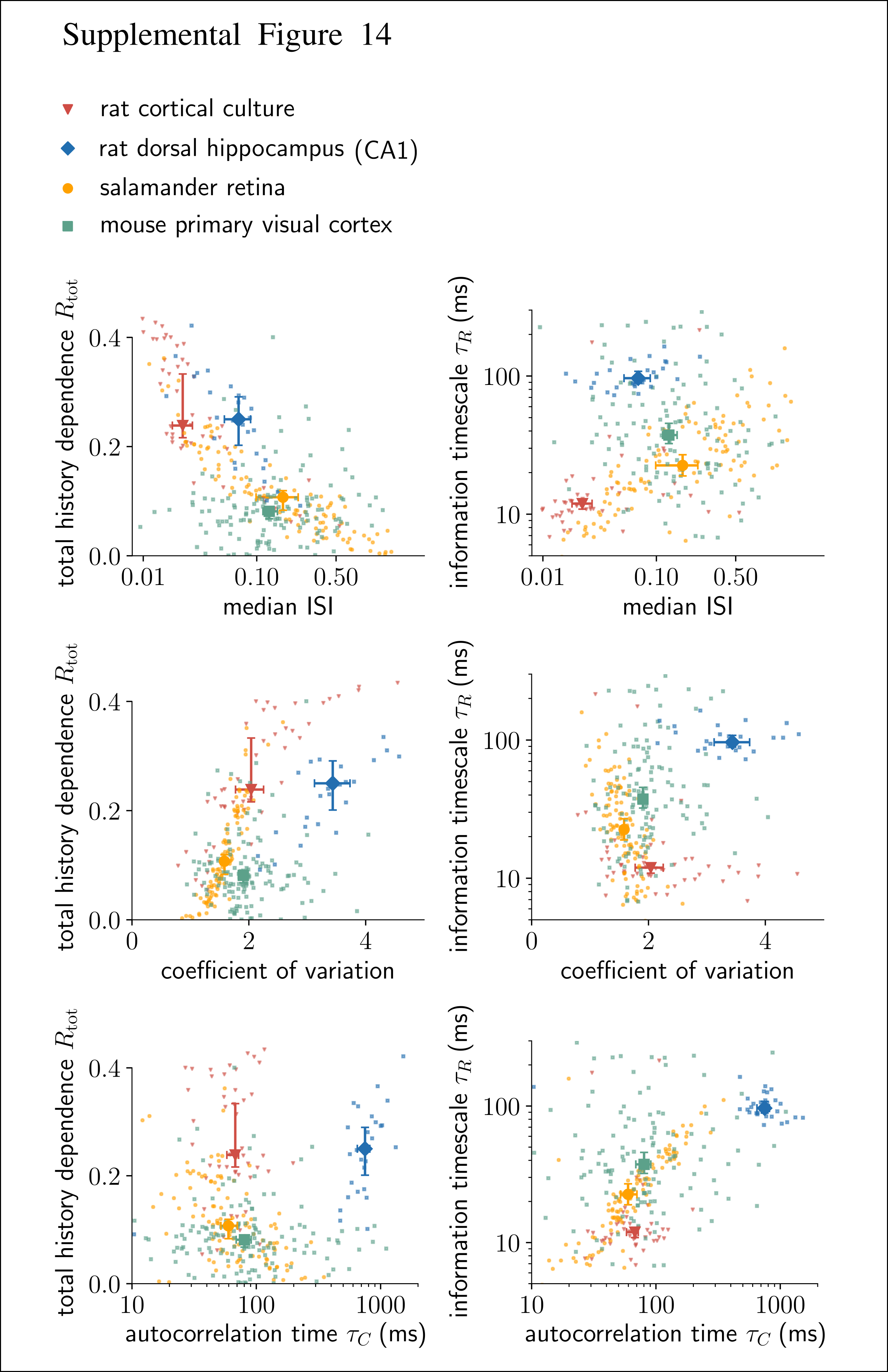

Supplement: S14 Fig — Estimates of the total history dependence Rtot tend to decrease with the median interspike interval (ISI), and to increase with the coefficient of variation CV. This result is expected for a measure of history dependence, because a shorter median ISI indicates that spikes tend to occur together, and a higher CV indicates a deviation from independent Poisson spiking. In contrast, the information timescale τR tends to increase with the autocorrelation time, as expected, with no clear relation to the median ISI or the coefficient of variation CV. However, the correlation between the measures depends on the neural system. For example in retina (n = 111), Rtot is significantly anti-correlated with the median ISI (Pearson correlation coefficient: r = −0.69, p < 10−5) and strongly correlated with the coefficient of variation CV (r = 0.90, p < 10−5), and τR is significantly correlated with the autocorrelation time τC (r = 0.75, p < 10−5). In contrast, for mouse primary visual cortex (n = 142), we found no significant correlations between any of these measures. Results are shown for the Shuffling estimator with dmax = 5, and T0 = 10 ms. Errorbars indicate median over sorted units and 95% bootstrap confidence intervals on the median. (TIF) [file pcbi.1008927.s014.tif]

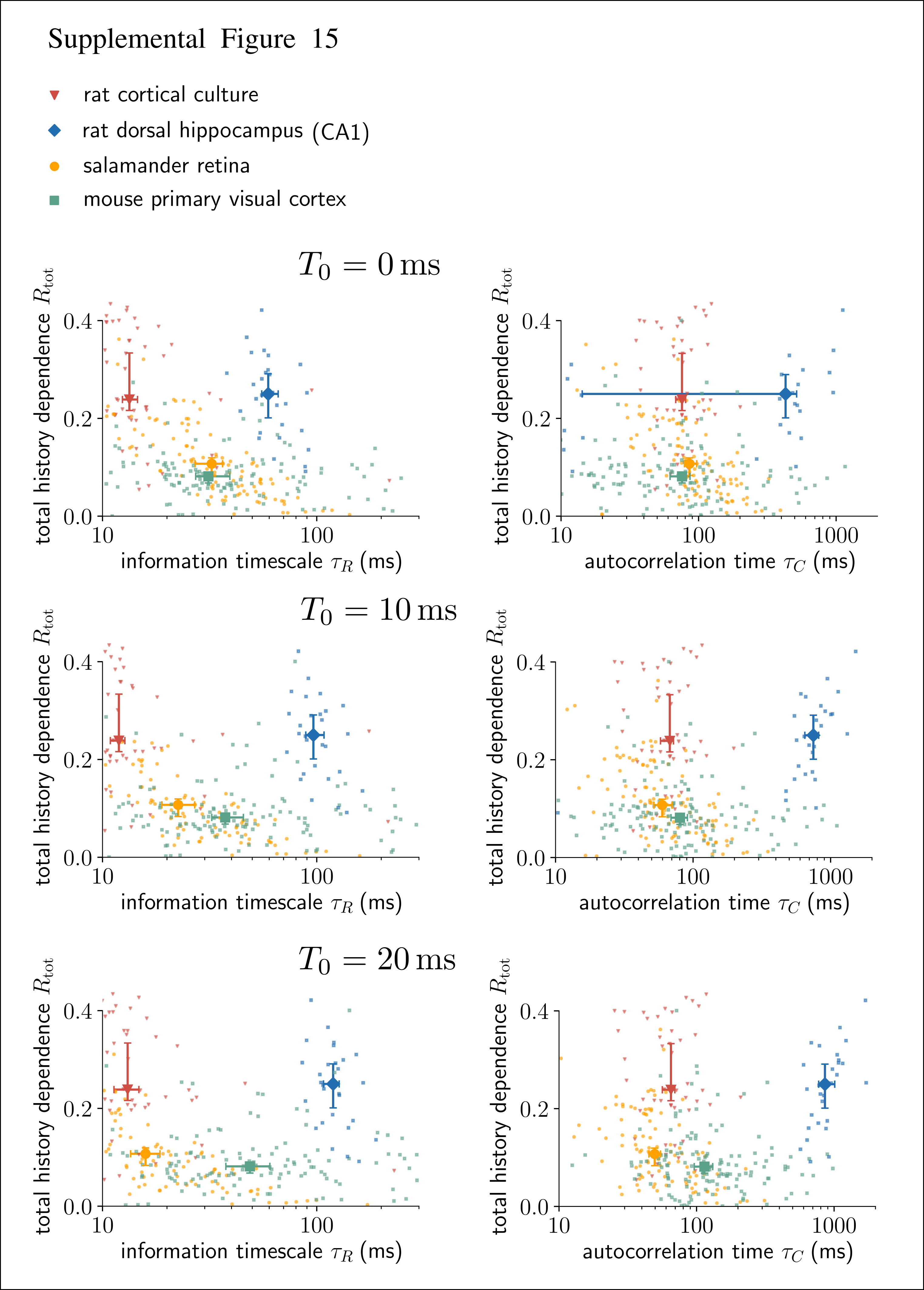

Supplement: S15 Fig — By only considering gains ΔR(T) for past ranges T > T0 when computing the information timescale τR, short-term effects that are related to the refractory period and different firing modes are excluded. The higher T0, the higher is the distance in the median τR between systems (especially between salamander retina and mouse primary visual cortex). This is because both timescales τR and τC increase with T0 for CA1 and primary visual cortex, whereas they decrease for retina. The same holds for the autocorrelation time τC, where only time lags T > T0 were considered when fitting an exponential decay to the autocorrelograms. Note that if the decay is perfectly exponential, then T0 does not affect the results. Estimates of Rtot and τR are shown for the Shuffling estimator with dmax = 5. Errorbars indicate median over sorted units and 95% bootstrap confidence intervals on the median. (TIF) [file pcbi.1008927.s015.tif]

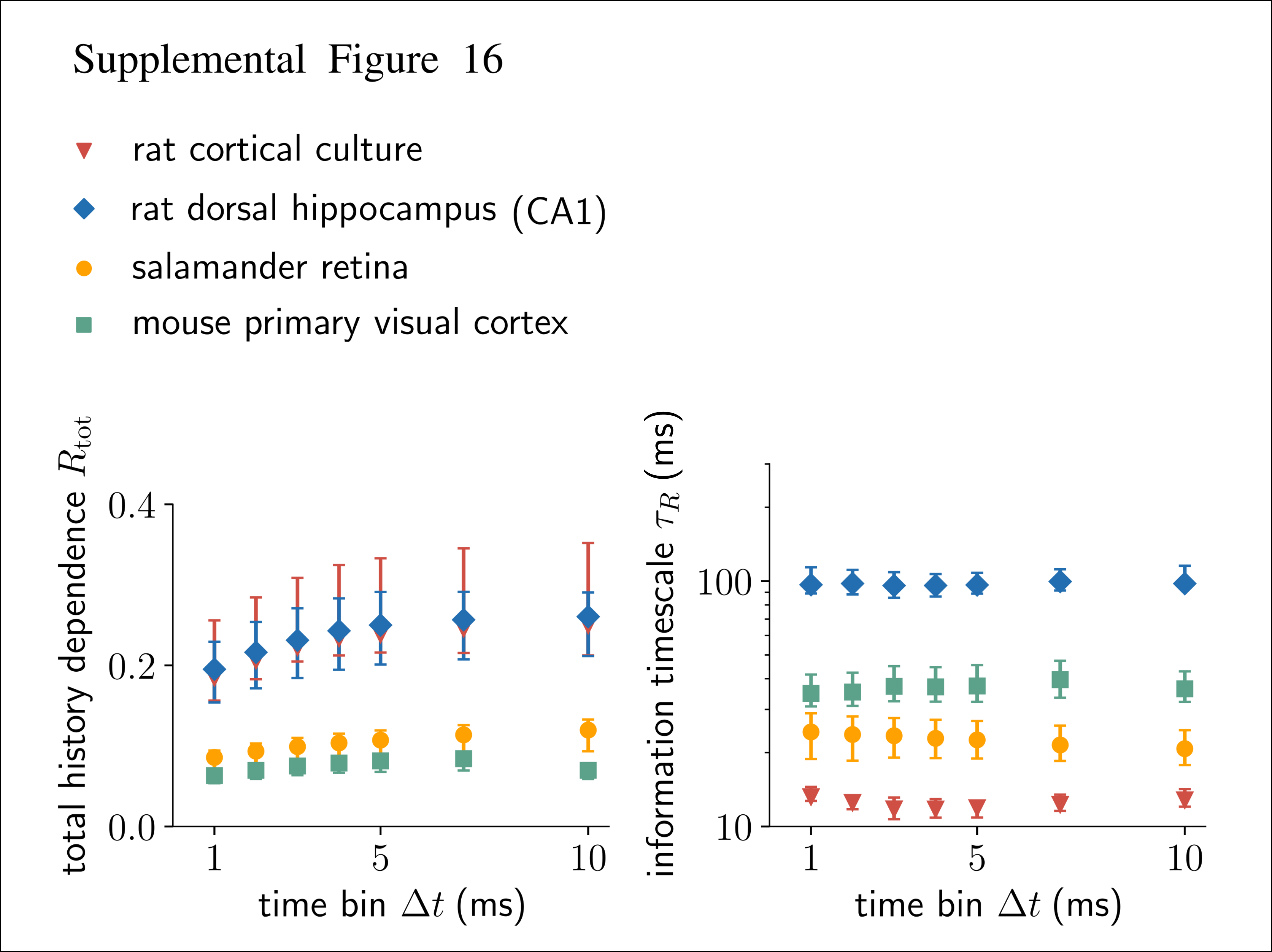

Supplement: S16 Fig — The choice of the time bin Δt of the spiking activity has little effect on the information timescale τR, whereas the total history dependence Rtot decreases for small time bins Δt < 5ms. This is consistent across experiments. The smaller the time bin, the higher the risk that noise in the spike emission reduces the overall predictability or history dependence in the spiking, whereas an overly large time bin holds the risk of destroying coding relevant time information in the spike train. Thus, we chose the smallest time bin Δt = 5 ms that does not yet show a substantial decrease in Rtot. We do not plot results for higher Δt, because for higher Δt we observed many instances of multiple spikes in the same time bin. Results are shown for the Shuffling estimator with dmax = 5, and T0 = 10 ms. Errorbars indicate median over sorted units and 95% bootstrap confidence intervals on the median. (TIF) [file pcbi.1008927.s016.tif]
