# Supplementary material for: Embedding optimization reveals long-lasting history dependence in neural spiking activity
Source: PLoS Comput Biol. 2021 Jun 1;17(6):e1008927. doi: 10.1371/journal.pcbi.1008927 (PMC8205186; doi:10.1371/journal.pcbi.1008927)
Supplement: Attachment — Submitted filename: response_to_reviewers.pdf [file pcbi.1008927.s017.pdf]

L. Rudelt, D. G. Marx, M. Wibral, V. Priesemann

Max-Planck-Institute for Dynamics and Self-Organization
Am Faßberg 17
D-37077 Göttingen
✉ lucas.rudelt@ds.mpg.de

Göttingen, March 9, 2021

Resubmission of our manuscript to *PLOS Computational Biology*

Dear editors, dear reviewers,

we thank you for your editorial consideration and the very helpful comments. Please find enclosed a point-to-point response to the reviewer's comments, and a manuscript file with changes highlighted in colour.

In brief, the main improvements in the revised version of the manuscript comprise (1) a clarification of our approach with respect to previous approaches that quantify temporal dependence in neural spike trains, (2) we compare our approach to classical measures, and show more simulated example neurons to illustrate the properties of the new approach, and (3) we revised the definition of the timescale.

In more detail, first, we carve out more clearly that our measure of history dependence assesses the window over which unique predictive information is accumulated; in contrast to e.g. autocorrelation, which assesses how long—potentially redundant—past information can still be read out. Moreover, the conventional estimate of timescale, the autocorrelation time, mixes the effects of strength and timescale of history dependence. In contrast, these are disentangled with our method.

Second, as proposed, we compare the novel measure on the example data sets to other well-established statistics, such as the median interspike interval, the coefficient of variation and the autocorrelation time. Moreover, we demonstrate its properties at a range of simulated model neurons, including the Izhikevich neuron. Third, we replaced the temporal depth of history dependence by a measure of a generalized timescale, which is equivalent to the autocorrelation time, but can also be applied to our measure of history dependence. With its similarity to the autocorrelation time, it facilitates the comparison to past work. In addition, this measure of timescale is more robust to the recording length, and thus further improves quantification.

With grounding our work in a more familiar terrain, and by introducing the robust measure of timescale, we could improve the clarity of our manuscript and method.

We thank you very much for your editorial consideration and are looking forward to your reply,

Lucas Rudelt & Viola Priesemann

Reviewer 1 This paper proposes new metrics for measuring history dependence in neural spike trains, and uses a particular coarse-graining in combination with existing entropy/mutual information estimation methods to estimate this metric for a range of neural spike trains. The authors then try to draw conclusions about their estimated metrics for various real neural spike trains.

The methods aspect of this seems relatively sound. I do have a suggestion for the authors, though, in terms of presentation: I'd put the vast majority of the methods in the Methods rather than the Results section. Basically, the discussion of the curse of dimensionality and the Data Processing Inequality in various forms (large number of bins is curse of dimensionality, can lead to overestimation and small number of bins yields lower MI due to Data Processing Inequality) seem to me to be well-worn statistical ground and not worthy of so much of the Results section.

> Thank you for your summary and your helpful comments. Indeed, we agree with you and had similar discussions during the writing process. However, since the article is aimed at a broad readership that might not be familiar with the issue of over- or underestimation, we found it important to illustrate it here. To incorporate your feedback in the revised manuscript, we included a statement at the beginning of the benchmark results section that encourage readers familiar with the concepts to skip this part (lines 341–344 in the new manuscript).

I'd also emphasize more that your main contribution to estimation of these information quantities is a particularly clever coarse-graining that assumes the recency hypothesis.

> Thank you for this suggestion. We clarified this contribution for the estimation by mentioning it explicitly in the abstract. The relevant passage reads
> "To still account for the vastly different spiking statistics and potentially long history dependence of living neurons, we developed an embedding-optimization approach that does not only vary the number and size, but also an exponential stretching of past bins."
>
> However, we would also like to point out that while our approach is based on established estimators, the way the approach uses them for regularization during the embedding optimization is novel and key to the estimation. As you point out, the coarse-graining with the recency hypothesis is an additional important step, but the approach could be used to optimize any other embedding model.

But that's not my main worry. I'm mainly worried that the metric isn't necessarily the right one for the job. On the chopping block is not just your $R(T)$ (which I would not call a redundancy, but rather just a version of the predictive information divided by H) and T_D (which I have a few comments on later), but also the autocorrelation function (which you discard, for reasons that make sense) and the predictive information (which you essentially have a

version of in your numerator, but see Nemenman et al) and all the information measures in "Anatomy of a Bit" by Ryan James et al.

> Thank you very much for pointing this out. About the predictive information, we regularly refer to it using both terms, predictable or redundant information. It depends on the decoder perspective, whether the information is used or not. In addition, we refer now explicitly to these measures and the additional literature you quote. The relevant passage in the methods summary now reads
> "We quantify history dependence based on the mutual information
>
> $$I(\text{spiking}; \text{past}(T)) = H(\text{spiking}) - H(\text{spiking}|\text{past}(T)) \tag{1}$$
>
> between current spiking in a time bin $[t, t + \Delta t)$ and its own past in a past range $[t - T, t)$ (Fig 1B). Here, we assume stationarity and ergodicity, such that the measure is an average over all times t. This mutual information is also called active information storage [5], and is related to the predictive information [18,19]. It quantifies how much of the current spiking information $H(\text{spiking})$ can be predicted from past spiking."
>
> However, we want to stress that there are two important differences between $R(T)$ and the predictive information: First, $R(T)$ quantifies how well spiking *in the next time bin* can be predicted, similar to the active information storage [5], whereas predictive information also increases the range of predicted spiking with T. Therefore, $R(T)$ can have very distinct behavior as one increases T (for example, the asymptotic rate is zero, see next comment). We chose active information storage over predictive information, because we want to quantify how redundant or predictable the current spiking is, based on its immediate past. From a practical point of view, this quantify is also easier to estimate, because only the past range T has to be embedded.
> Second, we normalize the mutual information by the spiking entropy. This is a crucial step to obtain a measure of statistical dependence, instead of information, similar to the correlation coefficient that normalizes covariance by the variance of the process. See below for more details where we discuss this in light of our novel results.
> Finally, we would like to stress that the main goal here was not to introduce a new information theoretic measure, but to use existing tools from information theory to address a problem that was previously only tackled using measures like the autocorrelation. However, in order to do so, we find it necessary to normalize by the entropy.

Based on my experience playing with these metrics, I'd say the following: – it is likely that T_D will grow with the size of your data set, and so what's really relevant is the rate of growth; that may be a better way to distinguish between different time series;

Your are right, the previous measure of temporal depth T_D was highly sensitive to the size of the data set, which we showed in the old S2 and S3 Figs. Therefore, we revised this measure completely. We now define the information timescale τ_R, which is more robust with respect to data size (see new S2 and S3 Figs), and its definition has a nice analogy to the autocorrelation time. However, we feel that you are referring to an asymptotic rate of growth $R(T)/T$ as one lets $T \to \infty$, similar to the predictive information in [19]. In the case of $R(T)$, this rate will always be zero, because $R(T)$ (with or without normalization) is bounded by one (or the spiking entropy; see previous comment). Thus, no such rate of growth can be defined for this measure.

– it is likely that R(T) has some weird behavior with the time bin size for the present neural patterning that has not yet been discussed and should be;

We added a supplementary figure (S16 Fig) that shows the dependence of $R(T)$ on the time bin size for the experimental data. While the information timescale τ_R is quite insensitive to the choice of Δt, the total history dependence decreases for small Δt. We added a passage in the methods summary where we discuss and explain our choice of $\Delta t = 5\,\mathrm{ms}$, which reads
"Finally, all the above measures can depend on the size of the time bin Δt, which discretizes the current spiking activity in time. Too small a time bin holds the risk that noise in the spike emission reduces the overall predictability or history dependence, whereas an overly large time bin holds the risk of destroying coding relevant time information in the neuron's spike train. Thus, we chose the smallest time bin $\Delta t = 5\,\mathrm{ms}$ that does not yet show a decrease in history dependence (S16 Fig)."

– I still have no idea how or if either $R(T)$ or T_D (data set size) capture anything related to history dependence.

To clarify this, we would like to point you to the new first section in Results, as well as Figs 1, 3, 4, and S14 Fig that clarify the difference between $R(T)$ or τ_R and the autocorrelation time, time-lagged mutual information and the total mutual information (R_{tot} without normalization). For more details see below.

Before I recommend acceptance, I would ask for simulations of an Izhikevich neuron that can adopt different neuron types. The strawmen, in my opinion, should be first the autocorrelation function and then the predictive information. I believe that information measures of time series can reveal the type of neuron or aspects of how it behaves, but I don't see why I should switch from using the predictive information to using $R(T)$ or its relative T_D. What am I getting from $R(T)$ that I'm not getting from predictive information? What is the intuition behind introducing this new measure? What do the authors even mean by "history dependence"? If I am to normalize something like predictive information by single symbol

entropy, as the authors do here, what neural spike train do I now correctly classify as having long history dependence that I before believed had little history dependence? As I am missing this intuition from the paper, I cannot recommend acceptance– yet.

> Thank you very much for this comment. First of all, we added the analysis of the Izhikevich neuron, together with the GLIF and a stochastic branching process, as comparison (Fig 4). The history dependence, and the correlation or lagged mutual information clearly show distinct behavior. In addition, we analyzed a binary autoregressive process, where we could control the firing rate via an uncorrelated, external input, as well as the strength and temporal depth of past dependencies in the process (Fig 3). We find that the total history R_{tot} correctly captures an increase in the strength m of past dependencies, whereas the information timescale τ_R is only sensitive to the temporal depth of the process. In contrast, the two aspects are mixed in the autocorrelation time.
>
> The example also addresses your question why the *normalized* mutual information or redundancy $R(T)$ is the right measure for our purpose. The mutual information is proportional to the spiking entropy, which depends crucially on the time bin, as well as the neuron's firing rate. As a consequence, the total mutual information increases strongly with increasing strength of *uncorrelated* inputs, whereas R_{tot} stays almost unaffected, or rather decreases (Fig 3B). Thus, the mutual information cannot clearly distinguish between an increase in input, or history dependence. In addition, we found that the total mutual information is correlated with the firing rates of the neurons, whereas the normalization allows to compare history dependence in neurons with vastly different firing rates (S13 Fig).

Smaller things:
– I would not say that this measure of history dependence has anything to do with the efficient coding hypothesis, which is more about how stimulus is transformed by a neuron so that the neuron has maximal entropy, or sometimes (depending on who's using the term) is about how mutual information between stimulus and neuron is close to the entropy of the neural activity;

> Thank you for this comment. As you point out, there are different formulations of the efficient coding hypothesis. We refer to the first formulation, where a stimulus is transformed by neurons so that they have maximal entropy – here by reducing temporal redundancy within a single spike train. We refer to this line of efficient coding in the introduction when we write "In classical, noise-less efficient coding, history dependence should be low to minimize redundancy and optimize efficiency of neural information transmission [1-3]."
>
> Temporal redundancy is quantified by R_{tot}, such that one can test for signatures of this kind of efficient coding using this measure of history dependence. All

of this, however, only makes sense if little noise is present, such that the stimulus information is close to the capacity $H(\text{spiking})$ of the neuron. In contrast, when significant noise is present, low history dependence can also be a signature of strong, uncorrelated noise, and cannot be attributed to the efficiency of the encoding. In such a case, additional analyses that assess the noisiness in the stimulus encoding are required. As first hint, we find in an ongoing follow-up project on a data set where neurons are classified as having a significant or no significant receptive field (which could be associated to noisiness of their encoding), that neurons with no significant receptive field actually have higher R_{tot}, consistently across different visual areas (not published yet).

– I would add some words on when your embedding method is likely to fail, which is precisely when initial conditions really really matter and the recency hypothesis is inaccurate– e.g. network of Izhikevich neurons– and which (notably) some might call long-term history dependence.

We totally agree and mention possible limitations in the discussion:
"Finally, our approach uses an embedding model that ranges from uniform embedding to an embedding with exponentially stretching past bins—assuming that past information farther into the past requires less temporal resolution. This embedding model might be inappropriate if for example spiking depends on the exact timing of distant past spikes, with gaps in time where past spikes are irrelevant. In such a case, embedding optimization could be used to optimize more complex embedding models that can also account for this kind of spiking statistics."

However, we would like to emphasize that the degree of coarse-graining *is optimized* in our approach, so if the recency hypothesis is inaccurate, a uniform binning will be chosen. If more detailed knowledge about past dependencies is available, more specific embedding models could be optimized using our approach.

If the authors can convince me that their metric $R(T)$ and its relative T_D (which should really be some aspect of how T_D changes with recording length) contain useful information that stumps the predictive information, then I will happily recommend acceptance.

We hope that with the new figures and clarifications in the text we have convinced you of the usefulness of the analysis using R_{tot} and τ_R, and the differences to predictive information. In addition, for cases where the predictive information is of interest, the embedding optimization approach presented in this paper could facilitate its estimation, as is the case for $R(T)$.

Reviewer 2 This paper is a potentially important contribution to neuroscientific toolbox. The authors propose an extension of existing information theoretic approaches that allows for an unbiased estimation of a neuron's history dependence on temporal depth and history dependence. The paper presents a thorough approach to controlling bias and overfitting. Further, the method is applied to several open datasets and an intriguing finding is described. Finally, the code to apply the methods described in the paper is made available with thorough documentation.

> Thank you for the great summary, your helpful requests and comments and your support for improving the usability of the tool. To summarize our changes, we now extended to link to existing approaches, which will facilitate to put our results into context. We expanded and improved our work, first by introducing a measure of timescale that is technically much closer related to the autocorrelation time, second by extending the analysis to several example model neurons, and finally by including the additional analyses on the experimental data sets that you proposed. We think that now the advantages, the distinction from previous approaches, and also the limitations are now much clearer. In the following, we address each point you raised.

I am enthusiastic but have one minor concern and a few related requests for additional analyses described below. In addition, I made a pull request on Github that may help improve the usability of this tool; hopefully, the authors will build on it to include a few tests of the code. This is not a requirement for this review, but it would be great to see code coverage increase to $> 50\%$.

> Thank you very much for your contribution to the tool! That is really great! Building on your pull request, we have increased testing coverage to $86\,\%$.

The concern is the following. History dependence R depends on the entropy of current spiking conditional on the past, as well as on the entropy of current spiking. The average firing rate of a neuron changes its entropy; presumably, this is the reason that entropy of current spiking is in the denominator. In theory, the product does not depend on the neuron's average firing rate; however, it would be nice to get a demonstration that R_{tot} or T_D do not vary as a function of the GLIF neuron's average firing rate, median ISI, or CV. More importantly, I'd like to see a scatterplot of these quantities vs R_{tot} and T_D in the datasets from Fig. 5.

> We conducted the proposed analyses on the data sets and included them in S13 and S14 Figs. We have also added a paragraph in the results section that analyzes the relation between R_{tot} or τ_R and the median ISI, CV or autocorrelation time. The paragraph reads
> "To better understand how other well-established statistical measures relate to the total history dependence R_{tot} and the information timescale τ_R, we show R_{tot} and τ_R versus the median interspike inteval (ISI), the coefficient of variation

$C_V = \sigma_{\text{ISI}}/\mu_{\text{ISI}}$ of the ISI distribution, and the autocorrelation time τ_C in S14 Fig. Estimates of the total history dependence R_{tot} tend to decrease with the median ISI, and to increase with the coefficient of variation C_V. This result is expected for a measure of history dependence, because a shorter median ISI indicates that spikes tend to occur together, and a higher C_V indicates a deviation from independent Poisson spiking. In contrast, the information timescale τ_R tends to increase with the autocorrelation time, as expected, with no clear relation to the median ISI or the coefficient of variation C_V. However, the correlation between the measures depends on the recorded system. For example in retina ($n = 111$), R_{tot} is significantly anti-correlated with the median ISI (Pearson correlation coefficient: $r = -0.69$, $p < 10^{-5}$) and strongly correlated with the coefficient of variation C_V ($r = 0.90$, $p < 10^{-5}$), and τ_R is significantly correlated with the autocorrelation time τ_C ($r = 0.75, p < 10^{-5}$). In contrast, for mouse primary visual cortex ($n = 142$), we found no significant correlations between any of these measures. Thus, the relation between R_{tot} or τ_R and the established measures is not systematic, and therefore one cannot replace the history dependence by any of them."

Regarding the firing rate, we did not find any statistical influence on R_{tot} and τ_R (which replaces T_D) on the data sets (S13 Fig, bottom). In contrast, if one does not normalize by the entropy, one observes an increase in total mutual information with the firing rate (S13 Fig, top) - as expected. We also demonstrate the importance of the normalization in the new Fig 3B, where R_{tot} does not increase as one increases the strength of uncorrelated input, whereas the total mutual information does increase. The relevant passage in the results section reads
"The input strength h increases the firing rate and thus the spiking entropy $H(\text{spiking})$. This leads to a strong increase in the total mutual information $I_{\text{tot}} \equiv \lim_{T\to\infty} I(\text{spiking}; \text{past}(T))$, whereas the total history dependence R_{tot} is normalized by the entropy and does slightly decrease (Fig 3B). This slight decrease is expected from a sensible measure of history dependence, because the input is random and has no temporal dependence. In addition, input activations may fall together with internal activations, which slightly reduces the total history dependence."

Note however, that normalizing by the entropy does not mean that R_{tot} will not increase for higher firing rates. As an example, consider the GLIF model neuron, where higher firing rates will result in more past spikes that trigger the spike adaptation. In this case, the total history dependence increases with the rate. Yet, for the GLIF model, it is hard to tune parameters such that one can vary the firing rate, median ISI or CV in a controlled way; hence we did not include such an analysis in the paper.

If authors find no correlation there, it may be instructive to look for a different connection to

traditional statistics as described in the first section of Discussion. Surely, we won't find any perfect replacements for history dependence, but if T_D is loosely related to some function of autocorrelation, it will help ground researchers in more familiar terrain.

This is a great point, and a relation to previous measures of temporal dependence was clearly missing. We added a new introduction Figure (Fig 1) that clarifies the difference between $R(T)$ and measures of temporal dependence such as the autcorrelation $C(T)$ and the lagged mutual information $L(T)$. Moreover, we added two figures (Figs 3 and 4) that clearly show, for three different models, how $R(T)$ capture aspects of history dependence that are not captured by $C(T)$ or $L(T)$. Finally, we added plots that compare the autocorrelation time τ_C to R_{tot} and τ_R in S14 Fig, and added a scatter plot of R_{tot} versus τ_C in the Results section in Fig 7B (previously Fig 5). The relevant passage from the Results section reads
"Notably, total history dependence and the information timescale varied independently among recorded systems, and studying them in isolation would miss differences between recorded systems, whereas considering them jointly allows to distinguish the different systems. Moreover, no clear differentiation between cortical culture, retina and primary visual cortex is possible using the autocorrelation time τ_C (Fig 7B), with medians $\tau_C \approx 68\,\text{ms}$ (culture), $\tau_C \approx 60\,\text{ms}$ (retina) and $\tau_C \approx 80\,\text{ms}$ (primary visual cortex), respectively."

We also discuss these results in the first section of Discussion, where the relevant paragraph reads
"A key difference between history dependence $R(T)$ and the autocorrelation or lagged mutual information is that $R(T)$ quantifies statistical dependencies between current spiking and the *entire past spiking* in a past range T (Fig 1B). This has the following advantages as a measure of statistical dependence, and as a footprint of information processing in single neuron spiking. First, $R(T)$ allows to compute the total history dependence, which, from a coding perspective, represents the redundancy of neural spiking with all past spikes; or how much of the past information is also represented when emitting a spike. Second, because past spikes are considered jointly, $R(T)$ captures synergistic effects and dismisses redundant past information (Fig 4). Finally, we found that this enables $R(T)$ to disentangle the strength and timescale of history dependence for the binary autoregressive process. (Fig 3). In contrast, autocorrelation $C(T)$ or lagged mutual information $L(T)$ quantify the statistical dependence of neural spiking on a single past bin with delay T, without considering any of the other bins (Fig 1A). Thereby, they miss synergistic effects; and they quantify redundant past dependencies that vanish once spiking activity in more recent past is taken into account (Fig 4). As a consequence, the timescales of these measures reflect both, the strength and the temporal depth of history dependence in the binary autoregressive process (Fig 3)."

The following lists a few minor suggestions.

In most citations, the name of the journal is missing. Is this by design?

> Thank you very much for your attention, we fixed this issue. The problem was an incompatibility between the biblatex translator and the PLOS template.

In line 78, what does 'discrete past embedding of spiking activity' mean? Do you refer to a 'reduced representation' of the past, or the discrete nature of spiking data? I am trying to discern whether past embedding with binary data has been described in practical terms before.

> The 'discrete' refers to the 'reduced representation' of the past, because, from an information theoretic view, spikes hold an infinite amount of information due to the continuous nature of their time information. We have changed the term to 'binary past embedding', because this is a more precise description of the reduced representation that we use in this paper (even if multiple spikes occur in the same time bin, we represent them by 0 or 1).

In line 152, you may wish to say something like 'while minimizing the risk of overestimation'.

> Thank you for your suggestion, we adopted this formulation in the current manuscript.

Line 163 mentions errorbars, but none are visible in Fig. 2D. I think a different place in the paper mentions 2xSTD errorbars being too small to be visible, but does that come later?

> Thank you for pointing this out. The statement about errorbars not being visible was made in the corresponding results section. To avoid confusion, we included this statement also in the figure caption.

I am confused regarding the status of GLM in this paper. Line 337 justly points out its systematic underestimation of history dependence, while line 194 claims that the authors used GLM as ground truth for $R(T, d, \kappa)$. Please clarify.

> The difference in the two cases is that for the data sets, the model assumptions of the GLM are not met, whereas for the GLIF neuron, they are accurate. Therefore, in case of the GLIF neuron, we use the GLM as an analytical tool to benchmark the model-free estimation approach. On the data sets, however, the underlying model is not known. There, we use the model-free estimates to show that the GLM systematically underestimates history dependence, because the model assumptions do not fully agree with the data. To avoid confusion, we removed the sentence on how the ground truth for the GLIF was computed in the results section, and only refer to Materials and methods. There, we clarify that the GLM only serves as ground truth to this particular model, and not in general. The

corresponding passage reads

"We can thus fit a GLM to the simulated data for the given past embedding T, d, κ to obtain a good approximation of the corresponding true history dependence $R(T, d, \kappa)$. Note that this is a specific property if this model and does not hold in general. For example in experiments, we found that the GLM accounted for less history dependence than model-free estimates (Fig 6)."

In Fig 4, why are bootstrapping errorbars not centered around the median (bars' height)?

The bootstrapping errorbars or $95\,\%$ confidence intervals (here bootstrapped over different sorted units) are not centered around the median, because they do not assume a normal distribution. This is different from errorbars on estimates of $R(T)$, which result from "blocks of blocks" bootstrapping of the time series and assume a normal distribution.

When referring to results from extracellular recordings, it may be best to call the units identified through spikesorting "single units" rather than "neurons" to remind us that spikesorting is somewhat subjective.

This is a great suggestion. As some units are multi units, and others are single units, we now call them all "sorted units" or simply "units" throughout the manuscript.

In Fig 5, would it be possible to include a scatterplot of history dependence estimated from GLM?

The GLM is very costly to optimize, such that it is infeasible to estimate $R(T)$ as a function of T for all the sorted units, which is required to estimate the timescale τ_R. Therefore, we did not include such a scatterplot in Fig 7 (old Fig 5).

Please attempt to interpret the results of Fig 6 further. Why is it that single unit 3 has such a distinctive shape? What might this mean for the corresponding neuron's information processing? What follow-up would you suggest for researchers using your tool when they see shapes like these? Would inspecting autocorrelograms help? Include any diagnostic information you find helpful.

Thank you for digging deeper here. We extended the interpretation in the results section and followed your suggestion to add the autocorrelograms to Fig 8 (old Fig 6). The relevant passage in the results reads

> "In particular, sorted units display different signatures of history dependence $R(T)$ as a function of the past range T. For some units, history dependence builds up on short past ranges T (e.g. Fig 8A), for some it only shows for higher T (e.g. Fig 8B), and for some it already saturates for very short T (e.g. Fig 8C). A similar behavior is captured by the autocorrelation $C(T)$ (Fig 8, second row). The rapid saturation in Fig 8C indicates history dependence due to bursty firing, which can also be seen by strong positive correlation with past spikes for short delays T (Fig 8C, bottom). To exclude the effects of different firing modes or refractoriness on the information timescale, we only considered past ranges $T > T_0 = 10\,\text{ms}$ when estimating τ_R, or delays $T > T_0 = 10\,\text{ms}$ when fitting an exponential decay to $C(T)$ to estimate τ_C. The reason is that differences in the integration of past information are expected to show for larger T. This agrees with the observation that timescales among recorded systems were much more similar if one instead sets $T_0 = 0\,\text{ms}$, whereas they showed clear differences for $T_0 = 10\,\text{ms}$ or $T_0 = 20\,\text{ms}$ (S15 Fig)."

Related: What is the interpretation of a peak followed by decay in $R(T)$ as in Fig S7, row 2, middle two?

> This is a great question. A peak as in Fig S7, row 2 is an artefact of the estimation. It arises because the embedding-optimized estimator first captures relevant past dependencies as T increases. For larger T, however, these dependencies cannot be resolved due to the regularization and thus limited number of past bins. In theory, $R(T)$ is monotonously increasing with T, because more past information can only increase the mutual information. We explicitly use this knowledge when estimating R_{tot} and τ_R (see lines 846–866 in Materials and methods), such that this behavior has no negative impact on our key observables.

There is a typo in line 1298 and in caption to S4.

> Thank you, both has been updated.

The sentence that starts on line 1326 is too long. Also, it may be good to italicize 'blocks of blocks' here.

> Thank you, we adapted both.

Reviewer 3

Embedding optimization reveals long-lasting history dependence in neural spiking Activity

• Summary of the paper and novelties This work investigates how to reliably quantify the dependence of a single neuron's spiking on its own preceding activity, called history dependence. Previous studies used limited representations of past activity (the so-called past embedding) to estimate information theory-based measures. Here it is argued that a careful embedding of past activity is crucial. A novel embedding-optimization method is proposed here that optimizes temporal binning of past spiking to capture most of the magnitude and the temporal depth of history dependence. The new method is validated against simulated data of a LIF neuron model and empirical data from different databases that account for a large variety of spiking statistics.
• Strengths The main strengths of the work are:
- It is demonstrated that previous ad hoc embedding strategies are likely to capture much less history dependence, or lead to estimates that severely overestimate the true history dependence. The new method maximizes the estimated history dependence while avoiding overestimation.
- The new method is flexible enough to account for the variety of spiking statistics encountered in experiments.

> Thank you for the great summary and your positive and helpful comments. Below, you clearly pointed out the problems with estimating the temporal depth. Thereby, you stimulated us to come up with a different measures of a timescale. This new measure, which we call information timescale, is not only more robust with respect to the data size, but also allows a much better comparison to the timescale of autocorrelation. We also agree with you that the limitations of the approach should be discussed explicitly in the discussion, and have added additional paragraphs that address the limitations that you pointed out. Below, we address your points one by one.

• Weaknesses and suggestions A weak point of the work is that for spike trains with long temporal depths (e.g., larger than 3 seconds, as in Fig. 3 C), the temporal depth estimated by the optimization method is much smaller (630 ms). This is a critical point to discuss in terms of possible limitations to estimate the timescale of neural processing at different stages of the brain.

> This is a very important point that we now solved by improving our measure of the timescale of history dependence, the information timescale τ_R. This quantity is more robust with respect to the data size (see S2 and S3 Figs), while still resolving the differences in timescale between the data sets (Fig 7). Nonetheless, it remains challenging to estimate the correct timescale τ_R if the true timescale is so large as in the GLIF model neuron, where adaptation effects last up to 22s into the past (although the underestimation is much less than for the temporal depth). We added a paragraph in the discussion about this limitation. The relevant passage reads

"Moreover, there might be cases where a model-free estimation of the true timescale might be infeasible because of the complexity of past dependencies (S2 Fig, neuron with a 22 seconds past kernel). In this case, only ≈ 80 % of the true timescale could be estimated on a 90 minute recording."

However, to demonstrate that the method can in principle estimate the true timescale, we replaced the results on the GLIF model with 22s kernel with results on a truncated version of the adaptation kernel with 1s kernel (Fig 5), and moved the previous results to Supplementary information (S1 and S2 Figs).

Another drawback of the new optimization methods is that they perform worse on short recordings: the estimated history dependence is overestimated when applying BBC to recordings of 3 minutes (S1 Fig) and the estimated temporal depth is underestimated to half of the real temporal depth (S2 Fig). This aspect might be discussed in the paper, analyzing possible limitations on application of optimization techniques to experimental data of short length.

We totally agree with you. Originally, these limitations were only discussed in the practical guidelines at the end of Methods and Materials section. However, as these limitations are of key relevance to the embedding optimization and analysis of history dependence, we added two paragraphs on these limitations in the discussion. The relevant passages read

"In contrast, the generalized timescale can be directly applied to estimates of the history dependence $R(T)$ to yield the information timescale τ_R without any further assumptions or fitting models. However, we found that estimates of τ_R can depend strongly on the estimation method and embedding dimension (S12 Fig) and the size of the data set (S2 and S3 Figs). The dependence on data size is not so strong for the practical approach of optimizing up to $d_{\text{max}} = 5$ past bins, but still we recommend to use data sets of similar length when aiming for comparability across experiments."

and

"Another downside of quantifying the history dependence $R(T)$ is that its estimation requires more data than fitting the autocorrelation time τ_C. To make best use of the limited data, we here devised the embedding optimization approach that allows to find the most efficient representation of past spiking for the estimation of history dependence. Even so, we found empirically that a minimum of 10 minutes of recorded spiking activity are advisable to achieve a meaningful quantification of history dependence and its timescale (S2 and S3 Figs). In addition, for shorter recordings, the analysis can lead to mild overestimation due to over-optimizing embedding parameters on noisy estimates (S1 Fig). This overestimation can, however, be avoided by cross-validation, which we find to be particularly relevant for the Bayesian bias criterion (BBC) estimator."

Regarding the underestimation of the temporal depth, we would like to point out that the information timescale that we introduce in the revised version is more robust to underestimation (new S2 Fig).

Some minor suggestions:
- Line 60: Could you comment on why the time bin of current spiking is chosen to be 5 ms?

This is an important question, and we have added a part to the Methods summary that explains how we chose the time bin, which we also support by a comparison of the experimental results for different choices of time bins (S16 Fig). The relevant passage reads
"Finally, all the above measures can depend on the size of the time bin Δt, which discretizes the current spiking activity in time. Too small a time bin holds the risk that noise in the spike emission reduces the overall predictability or history dependence, whereas an overly large time bin holds the risk of destroying coding relevant time information in the neuron's spike train. Thus, we chose the smallest time bin $\Delta t = 5\,\text{ms}$ that does not yet show a decrease in history dependence (S16 Fig)."

- Fig. 1 it is included in the Methods summary but is not well described in the text. Either move it to Methods, or further explain it here. In the figure caption, please provide more details of the figure, e.g., explain what is ML, NSB and BBC.

Thank you. We moved the figure to the Methods (now Figure 10), and expanded the figure caption.

- Fig S1 and paragraph between lines 272 and 286: how is each half of the data selected for cross-validation? Are multiple rounds of cross-validation performed using different partitions (in this case different halves) of the data?

We chose the most simple solution and literally take the first half of the data for the optimization of embedding parameters, and the second half for the optimization. Only one round of cross-validation is performed. What matters is that the set of embedding parameters is optimized on a different data set than the data set that is used to estimate $R(T)$. We edited the results paragraph and the figure caption to make this point more clear.

- Fig 4C: why BBC is computed with d = 20, and shuffling with d = 5?

We agree that this selection of estimates might be confusing, and have added Shuffling with $d_{\text{max}} = 20$ to Fig 6C (old Fig 4C). Now, all estimates from Fig 6B,D that allow exponential embedding are shown.

- Fig S4 is not referenced in the text.

Thank you for pointing this out, S4 and S5 Figs are now referenced in the results section on the benchmark model in lines 350 and 424.

- Typo: “errorbars” instead of “error bars” (for example, in line 262).

  Thank you, this was fixed.

- The publication year is missing in references.

  Thank you for your attention, the issue is fixed in the current version of the manuscript.

Methods are written in an appropriate and informative way

The paper is well written and concepts are provided in a correct, clear and suitable way.
